# Serum Levels of TWEAK and Scavenger Receptor CD163 in Type 1 Diabetes Mellitus: Relationship with Cardiovascular Risk Factors. A Case-Control Study

**DOI:** 10.1371/journal.pone.0043919

**Published:** 2012-08-24

**Authors:** Gemma Llauradó, José-Miguel González-Clemente, Elsa Maymó-Masip, David Subías, Joan Vendrell, Matilde R. Chacón

**Affiliations:** 1 Department of Diabetes, Endocrinology and Nutrition, Hospital de Sabadell, Corporació Sanitària i Universitària Parc Taulí (Universitat Autònoma de Barcelona), Sabadell, Spain; 2 Centro de Investigación Biomédica en Red de Diabetes y Enfermedades Metabólicas Asociadas (CIBERDEM), Hospital Universitari Joan XXIII de Tarragona, Insitut Pere Virgili, Universitat Rovira i Virgili, Tarragona, Spain; University of Siena, Italy

## Abstract

**Objective:**

To test the usefulness of serum concentrations of tumor necrosis factor-like weak inducer of apoptosis (sTWEAK) and soluble scavenger receptor CD163 (sCD163) as markers of subtle inflammation in patients with type 1 diabetes mellitus (T1DM) without clinical cardiovascular (CV) disease and to evaluate their relationship with arterial stiffness (AS).

**Methods:**

Sixty-eight patients with T1DM and 68 age and sex-matched, healthy subjects were evaluated. Anthropometrical variables and CV risk factors were recorded. Serum concentrations of sTWEAK and sCD163 were measured. AS was assessed by aortic pulse wave velocity (aPWV). All statistical analyses were stratified by gender.

**Results:**

T1DM patients showed lower serum concentrations of sTWEAK (Men: 1636.5 (1146.3–3754.8) pg/mL vs. 765.9 (650.4–1097.1) pg/mL; p<0.001. Women: 1401.0 (788.0–2422.2) pg/mL vs. 830.1 (562.6–1175.9) pg/mL; p = 0.011) compared with their respective controls. Additionally, T1DM men had higher serum concentrations of sCD163 (285.0 (247.7–357.1) ng/mL vs. 224.8 (193.3–296.5) ng/mL; p = 0.012) compared with their respective controls. sTWEAK correlated negatively with aPWV in men (r = −0.443; p<0.001). However, this association disappeared after adjusting for potential confounders. In men, the best multiple linear regression model showed that the independent predictors of sTWEAK were T1DM and WHR (R^2^ = 0.640; p<0.001). In women, T1DM and SBP were the independent predictors for sTWEAK (R^2^ = 0.231; p = 0.001).

**Conclusion:**

sTWEAK is decreased in T1DM patients compared with age and sex-matched healthy subjects after adjusting for classic CV risk factors, although sTWEAK levels may be partially influenced by some of them. Additionally, T1DM men have higher serum concentrations of sCD163. These results point out an association between the inflammatory system and CV risk in T1DM.

## Introduction

Cardiovascular (CV) disease is the major cause of mortality in type 1 diabetes mellitus (T1DM) [1]. As much as 10% of premature coronary artery disease morbidity and mortality in the general population is due to T1DM, but conventional CV risk factors (CVRF) account for no more than 25% of the excess CV disease risk [2]. As a result, the pathophysiological mechanisms underlying CV events in T1DM are not completely understood.

In recent years, several inflammation-related plasma proteins have been recognised as predictors of CV disease in the context of atherosclerosis in different populations [3]. Thus, T1DM subjects, a score of TNF-α, IL-6 and CRP has been associated with CV disease [4], focusing attention on chronic inflammation mediated by cytokines in T1DM CV disease.

The tumor necrosis factor-like weak inducer of apoptosis (TWEAK) is a relatively new member of the TNF superfamily of cytokines with multiple biological functions, including stimulation of cell growth and angiogenesis, induction of inflammatory cytokines and, under some experimental conditions, stimulation of apoptosis [5]. TWEAK induces production of proinflammatory cytokines, proliferation and migration of cells present in atherosclerotic plaques and increases the expression of metalloproteinases that degrade the extracellular matrix [5]. However, TWEAK plasma levels are diminished in patients with carotid atherosclerosis [6], coronary and peripheral artery disease [7], [8] or in atherosclerotic associated diseases, such as type 2 diabetes (T2DM) or end-stage renal disease [9]. The mechanisms leading to lower TWEAK plasma levels in subjects with atherosclerosis remain poorly understood.

CD163 is a new potential TWEAK scavenger receptor [10]. It has previously been identified as the receptor which uptakes haptoglobin-haemoglobin complexes for the removal and metabolism of the potent antioxidant haemoglobin [11]. A soluble form of CD163 (sCD163) is a normal constituent in plasma and is generated by proteolytic cleavage of CD163 at the cell surface [12]. This receptor is now recognized as an immunomodulator of the atherosclerotic plaque, with pivotal anti-inflammatory and anti-atherogenic properties [13]. sCD163 levels are increased in patients with atherosclerosis [14], [15] or atherosclerosis associated diseases, such as T2DM [16].

Our group has recently demonstrated that low-grade inflammation is associated with arterial stiffness (AS) in T1DM [17]. AS is an early sign of atherosclerosis [18] and its study would be appropriate in the investigation of atherosclerotic mechanisms long before any CV event occurs. According to a recent consensus [19], the gold standard for measuring AS is aortic pulse wave velocity (aPWV), and it predicts CV events and mortality independently of classical CVRF in several populations [3].

Because of the great burden of atherosclerosis in T1DM and the suggested association of TWEAK and CD163 with inflammation and CV disease, we carried out a study to gain some insight into the contribution of TWEAK and CD163 in the pathogenesis of CV disease in T1DM. For this purpose, we have determined serum concentrations of soluble TWEAK (sTWEAK) and sCD163 and their relationship with CVRF and AS in a group of subjects with T1DM without clinical CV disease and compared with age and sex-matched healthy subjects.

## Methods

Sixty-eight patients with T1DM (34 men and 34 women) aged 18–65 years (type 1 was defined as an onset of diabetes before the age of 36 years and insulin treatment iniciated within one year of diagnosis), and 68 age- and sex-matched healthy subjects were included in our study. None of them had any condition associated with an inflammatory response (e.g. acute or chronic infectious diseases) or had received anti-inflammatory treatment in the previous 6 months. None of them had any clinical CV disease. T1DM patients were consecutively recruited from our outpatient clinic and all had at least one year of evolution. The control group was recruited from hospital staff members and their relatives and friends.

The study methods have previously been described in detail [17]. Briefly, the following information was recorded using a predefined standardized form: gender, age, diabetes duration, body mass index (BMI), waist-to-hip ratio (WHR), systolic and diastolic blood pressure (SBP and DBP) and mean arterial pressure (MAP) – defined as 1/3 SBP +2/3 DBP-, physical activity (IPAQ questionnaire), cigarette smoking, insulin dose or the use of any other drug treatment, and microvascular complications. After an overnight fast, venous blood samples were taken and aliquots of plasma and serum were stored at −80°C until processing. In women, all measurements were done during the follicular phase of the menstrual cycle. HbA1c, lipid profile, serum concentrations of high-sensitivity CRP (hsCRP), IL-6, soluble fractions of the TNF-α receptors 1 and 2 (sTNFαR1 and sTNFαR2), sTWEAK and sCD163 were measured.

To estimate insulin-resistance in T1DM patients, we used the formula proposed by Williams *et al*, which yields an estimate of the glucose disposal rate (eGDR) taking into account glycemic control, WHR and blood pressure (eGDR  = 24.31–12.22 (WHR) – 3.29 (Hypertension 0 = No; 1 = Yes) –0.57 (HbA1)) [20]. It has been validated against euglycemic-hyperinsulinemic clamp in a group of T1DM subjects clinically similar to the subjects evaluated in the present study. Lower eGDR scores are reflective of greater insulin-resistance.

The study protocol was approved by our hospital Ethics Committee (Clinical Research Ethics Committee of Hospital of Sabadell), and was conducted according to the principles of the Declaration of Helsinki. All subjects gave their written informed consent before participating in the study.

### Assessment of microvascular complications

Peripheral polyneuropathy was assessed through a previously described two-step protocol combining the 15–item MNSI questionnaire and a physical examination evaluation [21]. Retinopathy was classified according to the data from our department database. Subjects were classified into three groups according to the degree of retinopathy: no retinopathy, non-proliferative retinopathy or proliferative retinopathy. Nephropathy was evaluated by the measurement of urinary albumin excretion. Subjects with a urinary albumin/creatinine ratio (ACR) greater than 3.4 mg/mmol (or 30 mcg/mg) [22], or previously treated with converting enzyme inhibitors or angiotensin receptor blockers (for microalbuminuria or macroalbuminuria), were considered as having diabetic nephropathy.

### Assessment of arterial stiffness

#### Measurement of aortic pulse wave velocity (aPWV)

We measured brachial blood pressure three times with the subjects in a sitting position: the mean of the last two measurements was used in all calculations. Subjects rested supine and measurements were taken immediately after the determination of blood pressure according to the recommendations of the recent consensus on AS [19]. aPWV was determined by sequential applanation tonometry at the carotid and femoral arteries gated to a three-lead ECG using the SphygmoCor® device (SphygmoCor®; AtCor, Sydney, Australia). Time delay was calculated using a foot-of-the-wave method. The surface distance from supra-sternal notch to each recording site was measured. The total transit distance was calculated by subtracting the sternal notch to carotid distance from the sternal notch to femoral distance. aPWV was calculated using the total transit distance divided by the time delay. aPWV not achieving the automatic quality controls specified by the SphygmoCor® software were rejected. The mean of two aPWV measurements was taken for each subject for all calculations. Data were available for all the participants included in the study.

### Laboratory analyses

HbA1c was determined by high-performance liquid chromatography (Menarini Diagnostics, Firenze, Italy). Total serum cholesterol, triglycerides, HDL-cholesterol and LDL-cholesterol were measured using standard enzymatic methods. hsCRP was determined by immunonephelometry (Siemens, Munich, Germany). IL-6 was determined by ELISA (R&D Systems, Oxon, UK), as were sTNFαR1 (Hycultbiotech, Uden, The Netherlands) and sTNFαR2 (R&D Systems, Oxon, UK). sTWEAK and sCD163 were determined by Human Duoset ELISA (R&D Systems, Oxon, UK).

### Statistical Analyses

Data are presented as percentages, means (SD) for variables normally distributed or medians (interquartile range) for variables not normally distributed. All data were tested for normality using the Kolmogorov-Smirnov test. To improve skewedness and kurtosis, variables not normally distributed were log transformed. The analyses were performed in the whole population and stratified by gender (p for interaction T1DM*gender for TWEAK = 0.090 and for CD163 = 0.006). Differences between T1DM patients and controls were analysed using the χ^2^ test for comparisons of proportions, and unpaired t-tests or Mann-Whitney U tests for comparisons of quantitative variables as needed. We assessed the potential relationships between sTWEAK and sCD163 with CVRF, low-grade inflammation and AS through univariate, non-parametric correlations. Linear regression models were used to adjust for potential confounders. Variables for linear regression analyses were selected based on univariate correlation analyses and those variables known or likely to be associated with both parameters. Because inflammatory-related serum proteins were only measured once, the association (if any) of low-grade inflammation with sTWEAK and sCD163 would tend to be underestimated. To address this issue, a Z-score was calculated for each inflammatory-related serum protein evaluated as: (value in the individual minus mean value in the study population)/SD. Subsequently, the low-grade inflammation general score was calculated as (Z-score of hsCRP + Z-score of IL-6+ Z-score of sTNFαR1 + Z-Score of sTNFαR2)/4. The IBM SPSS Statistics (v. 19 for Mac; SPSS, Inc, IBM Company) was used for all calculations. All p values were two-sided and a p value <0.05 was considered statistically significant. Regarding the power of the study when analysing the subjects by separated according to the gender, we have enough sample to detect at least a 20% percent of variation in sTWEAK concentrations in men and women with type 1 diabetes and their respective controls (α = 0.05 and β = 10%).

## Results

### Baseline characteristics

Sixty-eight patients with T1DM and 68 age- and sex-matched healthy subjects (n = 136) were included in the study. Their clinical and analytical characteristics are shown in Tables 1 and 2 (men and women respectively). The baseline characteristics have previously been described [17].

**Table 1:** Clinical characteristics of study population (men).

Men	Controls (n = 34)	T1DM (n = 34)	p
Age (years)	35.6 (9.0)	36.5 (8.9)	0.963
Current smokers (n, %)	7 (20.6)	12 (35.3)	0.089
Physical activity (METS-min/week)	1395.0(779.6–2265.8)	1903.0(910.5–2776.5)	0.270
Family history of CHD (n,%)	2 (5.9)	2 (5.9)	1.000
Family history of T2DM (n, %)	5 (14.7)	7 (20.6)	0.525
Family history of T1DM (n, %)	0 (0)	2 (5.9)	0.493
Hypertension (n, %)	3 (8.8)	13 (38.2)	0.004
Dyslipidemia (n, %)	17 (50.0)	18 (52.9)	0.808
Diabetes duration (years)	-	14.00 (8.50–20.50)	-
Microvascular complications (n, %)	-	9 (26.5)	-
Retinopathy (n,%)	-	5 (14.7)	-
None (n, %)	-	29 (85.3)	-
Non proliferative (n, %)	-	4 (11.8)	-
Proliferative (n, %)	-	1 (2.9)	-
Nephropathy (n, %)	-	6 (17.6)	-
Peripheral polyneuropathy (n, %)	-	0 (0)	-
BMI (kg/m^2^)	25.0 (2.8)	26.1 (3.3)	0.138
Waist (cm)	91.0 (10.1)	90.6 (11.3)	0.862
WHR	0.90 (0.10)	0.91 (0.07)	0.532
Systolic blood pressure (mmHg)	124.9 (9.5)	131.7 (10.8)	0.008
Diastolic blood pressure (mmHg)	73.8 (8.0)	76.7 (7.0)	0.124
Mean arterial pressure (mmHg)	90.8 (8.1)	95.0 (7.3)	0.029
Fasting plasma glucose (mmol/L)	4.76 (0.57)	8.71 (3.76)	<0.001
Total Cholesterol (mmol/L)	5.08 (1.54)	4.74 (0.82)	0.260
Triglycerides (mmol/L)	0.88 (0.69–1.29)	0.83 (0.69–1.14)	0.585
HDL-Cholesterol (mmol/L)	1.31 (1.13–1.49)	1.31 (1.11–1.76)	0.560
LDL-Cholesterol (mmol/L)	2.81 (2.26–3.59)	2.61 (2.23–3.13)	0.213
HbA1c (%)	5.4 (5.1–5.5)	7.3 (6.6–7.9)	<0.001
ACR (mg/mmol)	0.39 (0.30–0.51)	0.28 (0.20–0.47)	0.125
aPWV (m/s)	6.3 (5.7–6.7)	6.9 (6.5–7.9)	<0.001
hsCRP (mg/L)	0.6(0.3–1.1)	1.2(0.5–2.9)	0.036
IL-6 (pg/mL)	0.3(0.2–0.6)	0.6(0.3–1.0)	0.002
sTNFαR1 (pg/mL)	1410(1113–2308)	2739(1748–3224)	<0.001
sTNFαR2 (pg/mL)	2060(1870–2365)	2774(2267–3064)	<0.001
sTWEAK (pg/mL)	1636.5(1146.3–3754.8)	765.9 (650.4–1097.1)	<0.001
sCD163 (ng/mL)	224.8(193.3–296.5)	285.0(247.7–357.1)	0.012

Data are given as percentages, mean (SD) or median (interquartile range). CHD, coronary heart disease; ACR, urinary albumin/creatinine ratio.

**Table 2:** Clinical characteristics of study population (women).

Women	Controls (n = 34)	T1DM (n = 34)	p
Age (years)	35.3 (11.4)	35.2 (11.2)	0.971
Current smokers (n, %)	9 (26.5)	12 (35.3)	0.536
Physical activity (METS-min/week)	1386.0 (770.3–2079.0)	1386.0 (672.8–1686.0)	0.442
Family history of CHD (n, %)	4 (11.8)	1 (2.9)	0.356
Family history of T2DM (n, %)	7 (20.6)	9 (26.5)	0.567
Family history of T1DM (n, %)	1 (2.9)	3 (8.8)	0.614
Hypertension (n, %)	0 (0)	4 (11.8)	0.114
Dyslipidemia (n, %)	17 (50)	14 (41.2)	0.465
Diabetes duration (years)	-	12.00 (6.75–18.00)	-
Microvascular complications (n,%)	-	7 (20.6)	-
Retinopathy (n,%)	-	5 (14.7)	-
None (n, %)	-	29 (85.3)	-
Non proliferative (n, %)	-	2 (5.9)	-
Proliferative	-	3 (8.8)	-
Nephropathy (n,%)	-	3 (8.8)	-
Peripheral polyneuropathy (n, %)	-	0 (0)	-
BMI (kg/m^2^)	23.0 (3.1)	25.3 (3.9)	0.009
Waist (cm)	76.3 (6.9)	80.0 (10.3)	0.092
WHR	0.80 (0.06)	0.81 (0.07)	0.529
Systolic blood pressure (mmHg)	116.3 (9.5)	118.3 (9.6)	0.379
Diastolic blood pressure (mmHg)	67.9 (7.8)	69.1 (7.9)	0.508
Mean arterial blood pressure (mmHg)	84.0 (7.9)	85.5 (7.5)	0.417
Fasting plasma glucose (mmol/L)	4.59 (0.48)	9.59 (3.55)	<0.001
Total Cholesterol (mmol/L)	5.18 (1.11)	4.82 (0.92)	0.146
Triglycerides (mmol/L)	0.72 (0.56–0.93)	0.70 (0.53–0.84)	0.484
HDL-Cholesterol (mmol/L)	1.77 (1.46–1.99)	1.80 (1.49–2.20)	0.377
LDL-Cholesterol (mmol/L)	2.78 (2.18–3.48)	2.47 (1.90–2.97)	0.056
HbA1c (%)	5.3 (5.2–5.4)	7.8 (7.1–9.1)	<0.001
ACR (mg/mmol)	0.38 (0.27–0.65)	0.47 (0.30–0.91)	0.315
aPWV (m/s)	6.0 (5.3–6.7)	6.4 (5.9–7.5)	0.023
hsCRP (mg/L)	0.9(0.4–2.8)	1.4(0.7–2.5)	0.447
IL-6 (pg/mL)	0.4(0.2–0.6)	0.6(0.3–1.2)	0.039
sTNFαR1 (pg/mL)	1917(1355–3295)	2262(1366–2978)	0.864
sTNFαR2 (pg/mL)	2215(1897–2700)	2295(2018–3006)	0.320
sTWEAK (pg/mL)	1401.0(788.0–2422.2)	830.1(562.6–1175.9)	0.011
sCD163 (ng/mL)	269.2(223.4–329.7)	246.7(197.4–285.0)	0.144

Data are given as percentages, mean (SD) or median (interquartile range). CHD, coronary heart disease; ACR, urinary albumin/creatinine ratio.

T1DM patients showed lower serum concentrations of sTWEAK in both men and women, when compared with their respective counterparts (Tables 1 and 2). In addition, T1DM men had higher serum concentrations of sCD163 compared with their respective controls. No differences were observed regarding circulating levels of this soluble receptor in women.

#### Association of sTWEAK and sCD163 with CVRF, low-grade inflammation, insulin-resistance, microvascular complications and arterial stiffness

In univariate correlations, sTWEAK correlated negatively with T1DM status, smoking, WHR, SBP and DBP, MAP, fasting glucose and HbA1c, IL-6 and with the low-grade inflammation score in the whole population and in men. In women, sTWEAK was inversely associated with T1DM status, SBP and DBP, MAP, fasting plasma glucose and HbA1c. In the whole population, sCD163 did not show any correlation with CVRF or low-grade inflammation. However, sCD163 was positively associated with T1DM status, HbA1c and sTNFαR2 only in men (Table 3). In T1DM men, sTWEAK was negatively associated with diabetic nephropathy (r = −0.520; p = 0.003) and positively with eGDR (r = 0.375; p = 0.038). In T1DM women, sCD163 correlated negatively with eGDR (r = −0.428; p = 0.012). Regarding aPWV, a negative association with sTWEAK (r = −0.443; p<0.001) was observed in men. However, this association disappeared after adjusting for age, diabetes status, BMI, MAP and total cholesterol. Additionally, no significant association between sTWEAK and aPWV was found in women (r = −0.182; p = 0.169). No association between sCD163 and aPWV was found either in men or women (Men: r = 0.046; p = 0.711. Women: r = −0.076; p = 0.539).

**Table 3:** Spearman correlation coefficients for the association between sTWEAK and CD163 with CVRF and low-grade inflammation.

		sTWEAK			sCD163	
	Whole population	Men	Women	Whole population	Men	Women
Age (years)	−0.129	−0.232	−0.044	−0.035	−0.204	−0.105
Diabetes mellitus (N/Y)	−0.500**	−0.677**	−0.333*	0.070	−0.309*	−0.178
Smoking (N/Y)	−0.263**	−0.425**	−0.105	−0.109	−0.017	−0.234
Physical activity (METS-min/week)	0.076	−0.054	−0.160	0.137	0.094	−0.161
BMI (kg/m^2^)	−0.154	−0.252	−0.146	0.106	−0.161	−0.069
Waist (cm)	0.009	−0.229	−0.033	0.114	−0.163	−0.062
WHR	−0.034	−0.332**	−0.020	0.079	−0.030	−0.185
SBP (mmHg)	−0.279**	−0.458**	−0.404**	0.016	−0.080	−0.155
DBP (mmHg)	−0.200**	−0.334**	−0.273*	−0.010	−0.035	−0.102
MAP (mmHg)	−0.262**	−0.399**	−0.381**	0.006	−0.042	−0.112
Total Cholesterol (mmol/L)	−0–094	−0.075	−0.083	−0.004	−0.046	−0.037
Triglycerides (mmol/L)	−0.066	−0.203	−0.011	0.095	−0.101	−0.038
HDL-Cholesterol (mmol/L)	−0.131	−0.109	−0.263	−0.162	−0.237	−0.117
LDL-Cholesterol (mmol/L)	−0.010	−0.104	−0.056	0.086	−0.053	−0.114
FPG (mmol/L)	−0.363**	−0.429**	−0.294*	−0.064	−0.024	−0.151
HbA1c (%)	−0.482**	−0.654**	−0.314*	0.080	−0.286*	−0.103
hsCRP (mg/L)	−0.151	−0.178	−0.098	−0.115	−0.043	−0.202
IL-6 (pg/mL)	−0.233*	−0.368**	−0.115	−0.145	−0.052	−0.246
sTNFαR1 (pg/mL)	−0.163	−0.203	−0.109	0.071	−0.060	−0.079
sTNFαR2 (pg/mL)	−0.199	−0.249	−0.015	0.153	−0.260*	−0.051
Low-grade inflammation Score	−0.343**	−0.461**	−0.236	−0.037	−0.097	−0.180

SBP, systolic blood pressure; DBP, diastolic blood pressure, MAP, mean arterial pressure; FPG, fasting plasma glucose. *p<0.05. **p<0.01.

#### Multivariate regression analyses

To evaluate the main predictors of sTWEAK and sCD163, multiple linear regression analyses were performed.

#### Analysis considering the whole population

In the whole population, T1DM status and SBP were the independent predictors for sTWEAK (R^2^ = 0.341; p<0.001). In men, the best multiple linear regression model showed that the main determinants of sTWEAK were T1DM status and WHR (R^2^ = 0.640; p<0.001). In women, T1DM status and SBP were the independent predictors for sTWEAK (R^2^ = 0.231; p = 0.001) (Table 4).

**Table 4:** Independent sTWEAK predictors for the whole population.

Whole population (R = 0.604, R^2^ = 0.341)	β	95% CI	p
Constant	-	3.536–4.731	<0.001
T1DM status	−0.396	−0.328 to −0.144	<0.001
SBP	−0.327	−0.013 to −0.004	<0.001

Men (R = 0.800, R^2^ = 0.640)	β	95% CI	p
Constant	-	3.758–5.500	<0.001
T1DM status	−0.543	−0.419 to −0.209	<0.001
WHR	−0.253	−1.908 to −0.277	0.010

Women (R = 0.481, R^2^ = 0.231)	β	95% CI	p
Constant	-	3.336–5.090	<0.001
SBP	−0.353	−0.018 to −0.004	0.004
T1DM status	−0.312	−0.327 to −0.045	0.011

Variables entered into this model: age, (gender), smoking, physical activity, hypertension (N/Y), dyslipidemia (N/Y), WHR, SBP, logTriglycerides, logLDL-cholesterol, T1DM status and logHb1Ac.

#### Analysis in T1DM cohort

In T1DM patients, the main predictors were SBP, retinopathy and HbA1c (R^2^ = 0.379; p = 0.007) (Table 5).

**Table 5:** Independent sTWEAK predictors for T1DM population.

log sTWEAK (R = 0.615, R^2^ = 0.379)	β	95% CI	p
Constant	-	3.560–6.102	<0.001
Retinopathy	−0.397	−0.263 to −0.036	0.012
logHb1Ac	−0.370	−2.322 to −0.194	0.022
PAD	−0.330	−0.018 to 0.000	0.039

Variables initially entered into this model: age, gender, smoking, physical activity, hypertension (N/Y), dyslipidemia (N/Y), BMI, WHR, SBP, DBP, logTriglycerides, logLDL-cholesterol, logHDL-cholesterol, retinopathy, nephropathy, logHb1Ac, and logeGDR.

## Discussion

Inflammatory/anti-inflammatory imbalance has gained attention as an active player in the increased CV risk that accounts in T1DM. In the present study, we have shown that sTWEAK is decreased in T1DM patients. Additionally, T1DM men have higher serum concentrations of sCD163, suggesting that these cytokines may contribute to the inflammatory events that accounts in T1DM.

In previous studies, decreased concentrations of sTWEAK has been described in patients with carotid atherosclerosis [6], coronary and peripheral arterial disease [7], [8] or in atherosclerotic associated diseases, such as T2DM or end-stage renal disease [9]. These studies are in agreement with the data observed in our cohort of T1DM patients, being a disease with high prevalence of CV risk. Thus, the presence of T1DM was a strong predictive determinant of circulating levels of sTWEAK. Similarly, the data observed with sCD163, a scavenger receptor being a potential target of TWEAK, indicated higher levels in T1DM men. High levels of this receptor are usually associated with a poor CV profile, the opposite of that observed for sTWEAK. Previous data have shown that sCD163 levels are increased in patients with atherosclerosis [14], [15] or atherosclerosis associated diseases, such as T2DM [16]. Nevertheless, this association was not maintained when controlling for classical CVRF in our cohort.

In our study, sTWEAK concentrations were negatively associated with blood pressure and glycemic metabolic control in both genders. Previous studies found that fasting plasma glucose was the only parameter correlated with sTWEAK whereas no significant associations were found with the rest of CVRF [6], [9]. Interestingly, we observed an inverse association between central obesity and serum concentrations of sTWEAK and also an inverse association between insulin-resistance (assessed as eGDR) and lower levels of sTWEAK. Therefore, both central obesity and insulin-resistance would be associated with a decrease in sTWEAK concentrations, as the rest of CV risk factors. These data are in accordance with a previous report by Kralisch *et al*. in which HOMA-IR and sTWEAK were inversely associated in T2DM patients on chronic hemodialysis [9]. In contrast, in our patients sCD163 was only marginally correlated with metabolic control (only in men) and with insulin-resistance in women, without any correlation with CVRF. Reported data in the literature on the relationship between sCD163 and CVRF are contradictory. Thus, some authors have reported a positive correlation between sCD163 and anthropometric measures or blood pressure [23] but others have failed to find such associations [14], [15].

Moreover, we have shown, for the first time, an inverse association between sTWEAK concentrations and microvascular complications. In T1DM, retinopathy was one of the main predictors of sTWEAK. In contrast, it has been previously reported that TWEAK can stimulate blood vessel formation in the rat cornea angiogenesis assay, but it is presently unknown whether this cytokine could play a role in the pathological angiogenesis associated with diabetic retinopathy [24]. An inverse relationship between serum levels and local concentration in the atherosclerotic plaques has already been described in patients with high CV risk [5]. These differences may indicate dual action regarding local or systemic activity. Whether sTWEAK could be a potential marker of diabetic chronic complications warrants further investigation in prospective studies.

The role of TWEAK may seem counterintuitive because TWEAK induces some inflammatory markers and increases the expression of metalloproteinases that degrade the extracellular matrix [5]. However, in contrast to this proinflammatory role, recent evidence suggests that TWEAK could attenuate the transition from innate to adaptative immunity [25], modulating the activity of other inflammatory cytokines, like TNFα. As such, previous studies have found a negative correlation between sTWEAK and inflammatory markers [26], [27]. In concurrence with these results, we found a negative correlation between sTWEAK and IL-6 and with the low-grade inflammation score. Conversely, sCD163 concentrations have been associated with inflammatory markers [14], [23], [28]. This inflammatory profile attributed to sCD163 was only marginally observed in our cohort, with a weak association with sTNFαR2 (only in men).

Whether sTWEAK and sCD163 play a role in the pathogenesis of atherosclerosis remains unclear. Previous data have shown that, intima-media thickness was negatively correlated with sTWEAK [6] and this association remained significant after adjusting for classic CVRF and inflammatory markers. In agreement with this report, we found a negative correlation between sTWEAK and AS (assessed as aPWV) but only in men, although this association disappeared after adjusting for potential confounders. Moreno *et al*. observed that intima-media thickness was positively associated with sCD163 concentrations with independence of traditional CVRF and inflammatory markers [14], [15]. However, we failed to find any association between AS and sCD163 in our cohort. Thereafter, we could not confirm the association between these two markers and AS; a marker of subclinical atherosclerosis, in T1DM patients. There are no previous data on the role of sTWEAK or sCD163 in the pathogenesis of AS. It is likely that carotid intima-media thickness reflects more advanced structural atherosclerotic changes in the arterial wall than AS [29]. Our population was younger than those included in previous studies reporting associations between intima-media thickness and sTWEAK or sCD163 concentrations. Perhaps these differences may explain the lack of association between AS and these markers in our study.

In prospective studies sTWEAK serum levels independently predicted an adverse prognosis in patients with chronic heart failure [30]. In addition, decreased sTWEAK level is an independent predictor of CV outcomes in patients with non-dialysis chronic kidney disease [31] and with peripheral arterial disease [27]. In contrast, elevated sTWEAK levels predicted an adverse short-term outcome in patients with ST-elevation myocardial infarction [32] and CV and all-cause mortality in hemodialysis patients [26]. Therefore, future prospective studies are needed to better understand the potential role of these markers in atherosclerosis and their prognosis value.

We are aware that one of the limitations of our study is the cross-sectional design; therefore any inference regarding causality cannot be made. In addition, its observational design does not allow us to ensure complete control of all the potential (unknown) confounding factors. The concentrations of sTWEAK and sCD163 were measured only once, which might underestimate the association between them and AS.

In conclusion, our study demonstrates sTWEAK is decreased in T1DM patients compared with age and sex-matched healthy subjects, even after controlling for classic CVRF. However, neither marker is associated with AS after adjusting for potential confounders.
